# A functional variant rs353292 in the flanking region of miR-143/145 contributes to the risk of colorectal cancer

**DOI:** 10.1038/srep30195

**Published:** 2016-07-22

**Authors:** Fang Yuan, Ruifen Sun, Lijuan Li, Bo Jin, Yanyun Wang, Yundan Liang, Guanglu Che, Linbo Gao, Lin Zhang

**Affiliations:** 1 Department of Immunology, West China School of Preclinical and Forensic Medicine, Sichuan University, Chengdu, Sichuan 610041, P.R. China; 2 Central Laboratory, Yunnan University of Chinese Traditional Medicine, Kunming 650500, Yunnan, P.R. China; 3 Department of Forensic Biology, West China School of Preclinical and Forensic Medicine, Sichuan University, Chengdu, Sichuan 610041, P.R. China; 4 Laboratory of Molecular and Translational Medicine, West China Institute of Women and Children’s Health; Key Laboratory of Obstetric & Gynecologic and Pediatric Diseases and Birth Defects of Ministry of Education; West China Second University Hospital, Sichuan University, Chengdu, Sichuan 610041, P.R. China

## Abstract

MicroRNA (miR)-143 and miR-145 have been identified as molecular regulators in cell proliferation, cell growth, clone formation, apoptosis, cell cycle, invasion, and migration. We previously found that rs353292 in the flanking region of miR-143/145 showed a high frequency in patients with colorectal cancer (CRC). To identify whether the rs353292 polymorphism is a risk factor for CRC, we conducted this study with larger samples. A total of 809 patients with CRC and 1005 gender matched controls were collected. The rs353292 polymorphism was genotyped by using TaqMan allelic discrimination. Dual luciferase reporter assay was carried out to measure the transcriptional activity. We found that the rs353292 polymorphism was associated with an increased risk for developing CRC in heterozygous comparison (adjusted OR = 1.70, 95% CI, 1.32–2.20, *P* < 0.001), dominant genetic model (adjusted OR = 1.62, 95% CI, 1.26–2.09, *P* < 0.001), and allele comparison (adjusted OR = 1.46, 95% CI, 1.16–1.84, *P* = 0.001). The rs353292 CT/TT carriers exhibited a lower expression of miR-143 compared to the CC carriers (*P* = 0.04). Moreover, the pGL3-rs353292T displayed a significantly lower luciferase activity than pGL3-rs353292C (*P* < 0.01). These findings indicate that the rs353292 polymorphism is functional and may be a risk factor for the development of CRC.

Colorectal cancer (CRC) is the third most common cancer and the fourth leading cause of cancer death around the world, with an estimated 1.2 million new cases and 600000 deaths per year^1^,^2^. The highest incidence of CRC is in developed countries of Europe, North America, and Oceania^1^. Recent reports revealed that the incidence is rapidly increasing in many Asian countries including China^3^. Even with the improvements in diagnosis, surgery, chemotherapy, and targeted therapy, the 5-year survival rate for CRC patients is estimated to be 62–64%^4^. Previous identified risk factors for CRC included obesity, smoking, dietary patterns, physical inactivity, and genetic factors^5^,^6^,^7^.

MicroRNAs (miRNAs) are a class of conservative endogenous non-coding RNA molecules, with 18–25 nucleotides in length, and have been found in variety of eukaryotic organisms^8^. Generally, miRNAs play a crucial role in cellular development, differentiation, proliferation, and apoptosis by binding to 3′ untranslated region (UTR) of target mRNA molecules, resulting in the degradation of mRNA and inhibition of protein translation. A series of studies have demonstrated that miRNAs act as potential “tumor suppressors” or oncogenes^2^,^9^. MiR-143 and miR-145, closely located on 5q33, are co-transcribed from a single promoter, resulting in a primary transcript containing both miRNAs^3^. In 2003, Michael *et al*. initially reported that the expression of both miRNAs were downregulated in colorectal neoplasia^10^. The two miRNAs are always involved in various cancer-related events including proliferation, invasion and migration, suggesting a potential possess of anti-tumorigenic activity. With regard to the regulation of miR-143/145, previous reports have shown that *KRAS*, Ras responsive element binding protein 1, serum response factor, and myocardin can regulate the expression of miR-143/145 by altering the transcription of target genes^11^,^12^,^13^.

Single nucleotide polymorphisms (SNPs), the most common type of genetic variation in human genome, was estimated to disrupt the process of miRNAs, and thus influence their expression. Recently, we discovered several SNPs in the flanking region of miR-143 and miR-145 cluster. Among them, rs353292, located in 673 bp upstream from the start site, was related to an increased risk of CRC^14^. Since the sample sizes are limited in our previous study^14^, in this study, we replicated the relationship between the rs353292 polymorphism and CRC risk by enlarging sample size. Moreover, dual luciferase reporter assay was used to examine whether the rs353292 polymorphism can affect the transcriptional activity.

## Results

### Association of the miR-143/145 rs353292 with risk of CRC

Demographic and clinical characteristics for all the subjects are listed in Table 1. The mean age (±SD) of the cases was 61.0 ± (12.7) years, which was higher than the controls (*P* < 0.001). There was no significant difference in gender distribution between cases and controls (*P* = 0.10).

The genotype distributions in the control group followed the HWE (*P* = 0.09). As shown in Table 2, a significant difference in genotype and allele distributions of the rs353292 was found between cases and controls. A significantly increased CRC risk was found to be associated with CT genotype compared to CC genotype (adjusted OR = 1.70, 95% CI, 1.32–2.20, *P* < 0.001). The CT/TT genotype carriers had a 1.62-fold increased risk of CRC compared to CC genotype carriers (adjusted OR = 1.62, 95% CI, 1.26–2.09, *P* < 0.001). Additionally, the rs353292T allele was associated with a significantly increased risk of CRC compared to C allele (adjusted OR = 1.46, 95% CI, 1.16–1.84, *P* = 0.001). Stratification analyses were done according to different variables, including differentiated status, clinical stage, and lymph node metastasis. Nevertheless, no significant association was detected between the rs353292 polymorphism and clinical features (Table 3).

### Association of the rs353292 with the expression of miR-143/145

**Figure 1:**
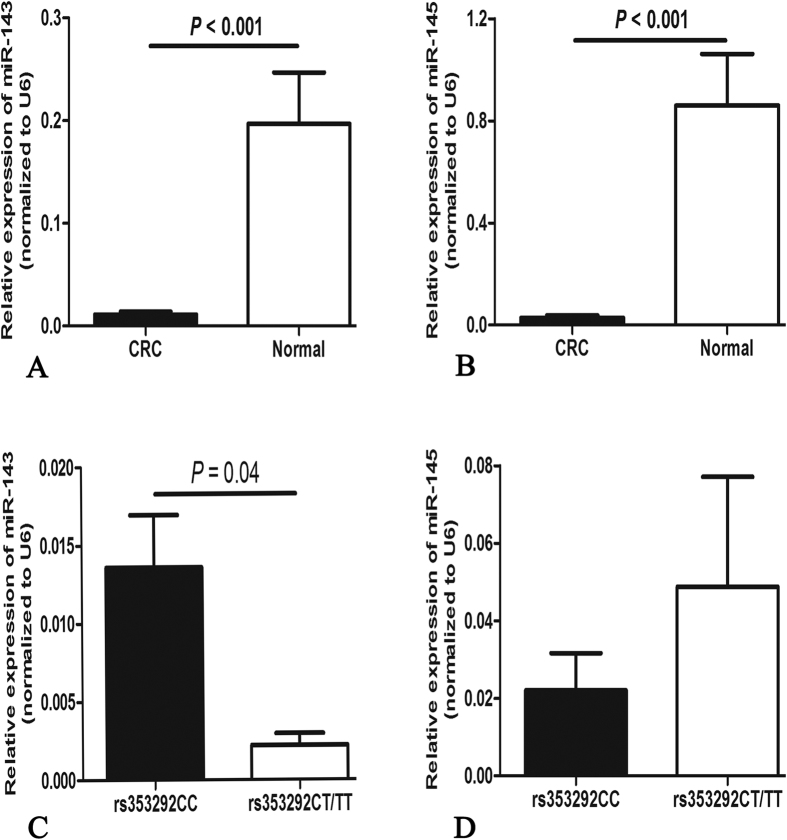
Relative expression of miR-143/145 in 71 CRC tumor tissues and normal tissues determined by qRT-PCR. Both miR-143 (**A**) and miR-145 (**B**) were found dramatically lower in CRC tissues (*P* < 0.001). MiR-143 was found significantly down-regulated in rs353292 CT/TT carriers (**C**). No significant relationship of miR-145 and the rs353292 polymorphism was found (**D**).

We detected the expression of miR-143 and miR-145 in both CRC tumors and noncancerous tissues. As shown in Fig. 1A,B, levels of both miR-143 and miR-145 in CRC tissues were dramatically lower than those in noncancerous tissues (*P* < 0.001). Furthermore, we analyzed the association between the rs353292 polymorphism and the expression of miR-143 and miR-145 in CRC tissues. We found that the expression of miR-143 in rs353292 CT/TT carriers was significantly lower than that in CC carriers (*P* = 0.04) (Fig. 1C). However, no significant correlation between the rs353292 polymorphism and the expression of miR-145 was found (Fig. 1D).

### Dual Luciferase Reporter Assay of the miR-143/145 rs353292 polymorphism

**Figure 2:**
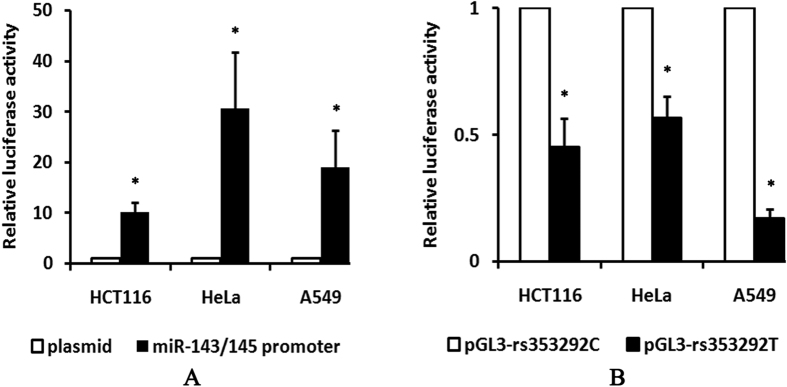
The relative luciferase activity in different cell lines. (**A**) After transfected with the plasmid containing the flanking region of miR-143/145, significantly higher luciferase activity was observed. (**B**) The pGL3-rs353292T led to a decrease in luciferase expression compared to the pGL3-rs353292C. Each experiment was done in triplicate and data are presented as mean ± SD. *P < 0.05.

After transient transfection of miR-143/145 flanking region into HCT116, HeLa, and A549 cells, the transcriptional activity was a significantly higher compared to empty plasmid (*P* < 0.01) (Fig. 2A). To further investigate the effect of the rs353292 polymorphism on the transcriptional activity, we constructed two plasmids: pGL3-rs353292T and pGL3-rs353292C. As shown in Fig. 2B, the pGL3-rs353292T showed a significantly lower luciferase activity than pGL3-rs353292C (*P* < 0.01). In HCT116 cells, the rs353292T displayed a 0.45-fold decreased activity compared to the rs353292C (*P* = 0.009). Similarly reduced luciferase activity was also observed in HeLa and A549 cells, with a fold change of 0.57 and 0.17 (*P* = 0.004 and *P* < 0.001, respectively).

## Discussion

We performed a case-control study evaluating the relationship between the rs353292 polymorphism in the flanking region of miR-143/145 and CRC risk. We found that the rs353292 polymorphism was associated with an increased risk for developing CRC in heterozygous comparison, dominant genetic model, and allele comparison. The rs353292 CT/TT carriers exhibited a lower expression of miR-143 compared to the CC carriers (*P* = 0.04). *In vitro* analysis showed that rs353292T exhibited a lower transcriptional activity compared to rs353292C. These findings suggest that the rs353292 is functional and may be used as a biomarker for the risk of CRC.

MiR-143 and miR-145 are located on chromosome 5q33.1 in human genome and are speculated to be cotranscribed as the same bicistronic unit^15^. These two miRNAs have been studied extensively and have been identified as “tumor suppressors” in varieties of cancer types, including CRC^8^,^16^,^17^,^18^,^19^,^20^,^21^,^22^,^23^,^24^,^25^,^26^,^27^,^28^. Downregulation of these miRNAs has been reported in both CRC tissues and CRC cell lines^16^,^17^,^18^,^20^,^21^,^22^,^23^,^24^,^25^,^28^. Such abnormal expression can regulate cell proliferation, cell growth, clone formation, apoptosis, cell cycle, invasion, and migration by targeting lots of genes such as metastasis-associated in colon cancer-1, insulin-like growth factor 1 receptor, DNA methyltranferase 3A, paxillin, toll-like receptor 2, fascin-1, and *KRAS*^16^,^17^,^18^,^20^,^21^,^23^,^24^,^25^. Furthermore, miR-143 can reduce viability and increase the sensitivity of CRC cells to 5-fluorouracil via extracellular-regulated protein kinase 5⁄nuclear factor-κB signaling pathway^26^. These findings indicate that miR-143 and miR-145 may be used as not only anti-oncogenic genes but also therapeutic targets for CRC.

Recently, several SNPs in the flanking region of miRNAs have been reported to alter individual’s susceptibility to cancer. Among them, an SNP rs4938723 in the promoter of pri-miR-34b/c was the mostly studied, which was reported to be related to the risk of a series of cancer types, such as colorectal cancer^29^,^30^, hepatocellular carcinoma^31^, gastric cancer^32^, renal cell cancer^33^, esophageal squamous cell carcinoma^34^, nasopharyngeal carcinoma^35^, papillary thyroid carcinoma^36^, and cervical cancer^37^. Other SNPs included rs10877887 in the promoter of let-7, rs2296616 in the promoter of miR-107, rs999885 in the promoter of miR-106b-25 cluster, and rs4705342 and rs4705343 in the flanking region of miR-143/145, which were reported to be associated with the risk of papillary thyroid carcinoma^38^, lung cancer^39^, gastric adenocarcinoma^40^, hepatocellular carcinoma^41^, prostate cancer^42^, and cervical squamous cell carcinoma^43^. In our previous study, we reported a new SNP rs353292 in the flanking region of miR-143/145 and the rs353292T allele was a risk factor for the development of CRC^14^. In this replication study, the same positive effect was also observed. In agreement with the association study, functional analysis revealed that the rs353292T allele had a reduced transcriptional activity *in vitro*. These findings indicate that the rs353292T allele may result in lower transcriptional activity and reduced level of miR-143, and eventually increase the risk of CRC.

Since DNA for the case group was extracted from paraffin-embedded specimens and DNA for the control group was isolated from peripheral blood samples, we cannot exclude the possibility of heterozygote between cases and controls. And thus confirmation studies should be performed using peripheral blood samples from both case and controls. Moreover, the mean age in the controls was lower than that in cases, which may result in unreliable results due to selection bias. After adjustment by age and gender, however, the positive results were still observed, suggesting the results may be reliable. Well-designed studies are of great importance to verify these findings, especially in other ethnic groups.

In conclusion, we provided the first evidence that the rs353292 in the flanking region of miR-143/145 may modulate transcriptional activity, and finally affect individual’s susceptibility to CRC. This work may help us achieve a deeper insight into SNPs in the flanking region of miRNAs relevant to the carcinogenesis of CRC. In the future, *in vivo* experiments using transgenic mice will be valuable to analyze the rs353492 polymorphism in the risk of CRC.

## Materials and Methods

### Ethics statement

The study protocol was approved by the Ethics Committee of the West China Second University Hospital (Ethics number: 028), and informed consent was obtained from all subjects. All the experiments were performed in accordance with the approved guidelines.

### Study population

A total of 809 paraffin wax-embedded CRC samples were consecutively recruited between January 2012 and October 2014 from the Affiliated Hospital of North Sichuan Medical College. Seventy-one fresh CRC tumor tissues and adjacent non-tumor tissues were collected from the Luoyang Central Hospital Affiliated to Zhengzhou University. All patients were diagnosed by histopathological confirmation. Clinical information was collected on age, gender, ethnicity, differentiated status, tumor size, lymph node metastasis, and distant metastasis, through a review of pathological and/or surgical reports. Clinical stage was estimated based on TNM classification. Patients with an evidence of personal or family history of inflammatory diseases or cancer in the intestine were excluded. The control group included 1005 healthy subjects from the hospital for a routine checkup at the same period. All the controls were gender and ethnicity matched to cases.

### Genotyping

For the case group, genomic DNA was extracted from paraffin wax-embedded sections; for the control group, genomic DNA was extracted from EDTA- anticoagulated peripheral blood, using commercial extraction kits (Qiagen, Hilden, Germany; Biobeke, Beijing, China). The rs353292 polymorphism was analyzed by using TaqMan allelic discrimination. The SNP assay was validated by sequencing about 5% of all samples, and the results were 100% concordant.

### RNA extraction and quantitative reverse transcription-PCR assay

Total RNA of tissue samples was extracted using TRIzol reagent according to the manufacturer’s instructions (Roche, Indianapolis, IN). 1 μg of total RNA was reverse-transcribed to cDNA according to the manufacturer’s manual of a commercial kit (Ribobio, Guangzhou, China). MiR-143/145 primers were purchased from Ribobio company (Guangzhou, China). Quantitative real-time PCR was performed using a SYBR green Master Mix (Qiagen, Hilden, Germany). The reaction conditions were as follows: 95 °C for 10 min, 95 °C for 10 sec, 60 °C for 20 sec, 70 °C for 10 sec (40 cycles). All reactions were performed in triplicate. Data were normalized using comparative threshold cycle method and U6 for normalization.

### Construction of reporter plasmid

Detailed information for constructing reporter plasmids was described in our previous study^43^. Briefly, the fragment containing rs353292T was synthesized by PCR with primers 5′-AGTGGTACCGCCGTGGAGAGTGGAATAGA-3′ (forward) and 5′-GTGAAGCTTCCAACTGACCAGAGATGCAG-3′ (reverse). The primers were designed to incorporate *Kpn*I and *Hind*III restriction sites, and the PCR product was cloned into the pGL3-basic vector (Promega, Madison, WI). The rs353292C allele was obtained by using a QuickChange Site-Directed Mutagenesis Kit (Stratagene, La Jolla, CA), and the mutation primers were as follows: 5′-GAAATATCCAGAAAATATACAGACAGATCTATAGAGATA-3′ (forward), and 5′-TATCTCTATAGATCTGTCTGTATATTTTCTGGATATTTC-3′ (reverse). All constructs were confirmed by direct sequencing (TsingKe, Chengdu, China). We named the generated reporter vectors of pGL3-rs353292T and pGL3-rs353292C, respectively.

### Transfection and dual luciferase reporter assay

HCT116, HeLa, and A549 cells were obtained from our laboratory. All the cell lines were plated on 24-well plates at a density of 1 × 10^5 ^cells/well. The cells were transiently transfected for 48 h with 2 μg pGL3-basic vector DNA or pGL3-rs353292T or pGL3-rs353292C using X-treme GENE HP reagent (Roche, Indianapolis, IN). According to the manufacturer’s protocol, 40 ng pRL-TK vector DNA (Promega, Madison, WI) was co-transfected as an internal control. The luciferase activities were measured on the Dual-Luciferase Reporter Assay System (Promega). The experiment was performed in triplicates and the results were reported as mean ± standard deviation (SD).

### Statistical analysis

The age distribution between the cases and controls was assessed using Student’s *t* test and the gender distribution was compared using chi-square test. Hardy-Weinberg equilibrium (HWE) was tested using chi-square test. The genotype and allele frequencies of the two groups were compared with chi-square test, and the relative risk was estimated by odd ratios (ORs) and 95% confidence intervals (CIs). The association between the rs353292 polymorphism and CRC was adjusted by age and gender using logistic regression model. The levels of luciferase reporter gene expression were examined using Student’s *t* test between different constructs. Statistical analyses were performed using the software Statistical Package for Social Science (SPSS) for Windows, version 19.0 (SPSS, Inc., Chicago, IL). A *P* value <0.05 was considered statistically significant.

## Additional Information

**How to cite this article**: Yuan, F. *et al*. A functional variant rs353292 in the flanking region of miR-143/145 contributes to the risk of colorectal cancer. *Sci. Rep.* **6**, 30195; doi: 10.1038/srep30195 (2016).

## Figures and Tables

**Table 1:** Demographics of the CRC Patients and Controls.

Variables	Cases n = 809 (%)	Controls n = 1005 (%)
Mean age (year)	61.0 ± 12.7	48.3 ± 15.0
Gender		
Male	482 (59.6)	560 (55.7)
Female	327 (40.4)	445 (44.3)
Tumor site		
Colon	244 (30.2)	
Rectal	563 (69.6)	
Both	2 (0.2)	
Differentiated status		
Well-moderately	419 (51.8)	
Poorly-undifferentiated	390 (48.2)	
Clinical stage		
I–II	479 (59.2)	
III–IV	330 (40.8)	
Lymph node metastasis		
Yes	291 (35.9)	
No	518 (64.1)	

**Table 2:** Association Between the rs353292 Polymorphism and Risk of CRC.

Genotypes	Cases n = 809 (%)	Controls n = 1005 (%)	Logistic regression (crude)		Logistic regression (adjusted^a^)	
			OR (95%CI)	*P* value	OR (95%CI)	*P* value
CC	601 (74.3)	841 (83.7)	1.00		1.00	
CT	201 (24.8)	152 (15.1)	1.84(1.46–2.33)	<0.0001	1.70(1.32–2.20)	<0.0001
CT/TT	208 (25.7)	164 (16.3)	1.77(1.41–2.23)	<0.0001	1.62(1.26–2.09)	<0.0001
Allele						
C	1403 (86.7)	1834 (91.2)	1.00		1.00	
T	215 (13.3)	176 (8.8)	1.60(1.29–1.97)	<0.0001	1.46(1.16–1.84)	0.001

^a^Adjusted for age and gender using the logistic regression model.

OR, odds ratio; CI, confidence interval.

**Table 3:** Association Between the rs353292 Polymorphism and Clinical Features of CRC Patients.

Clinical Features	Genotype Frequency (%)		OR (95% CI)	*P* value
Differentiated status	Well-moderately	Poorly-undifferentiated		
CC	318 (75.9)	283 (72.6)	1	
CT/TT	101 (24.1)	107 (27.4)	0.84 (0.61–1.15)	0.28
C	734 (87.6)	669 (85.8)	1	
T	104 (12.4)	111 (14.2)	0.85 (0.64–1.14)	0.28
Clinical stage	I–II	III–IV		
CC	357 (74.5)	244 (73.9)	1	
CT/TT	122 (25.5)	86 (26.1)	0.97 (0.71–1.34)	0.85
C	834 (87.1)	569 (86.2)	1	
T	124 (12.9)	91 (13.8)	0.93 (0.70–1.24)	0.62
Lymph node metastasis	Yes	No		
CC	213 (73.2)	388 (74.9)	1	
CT/TT	78 (26.8)	130 (25.1)	1.09 (0.79–1.51)	0.59
C	499 (85.7)	904 (87.3)	1	
T	83 (14.3)	132 (12.7)	1.14 (0.85–1.53)	0.39

OR, odds ratio; CI, confidence interval.
